# A Second-Generation Nanoluc-IL27 Fusion Cytokine for Targeted-Gene-Therapy Applications

**DOI:** 10.3390/bioengineering9020077

**Published:** 2022-02-16

**Authors:** Janelle Wesleyn Salameh, Shreya Kumar, Cosette Marie Rivera-Cruz, Marxa Leao Figueiredo

**Affiliations:** 1 The Department of Basic Medical Sciences, Purdue University, 625 Harrison St., West Lafayette, IN 47907, USA; wsalameh@purdue.edu (J.W.S.); kumar308@purdue.edu (S.K.); riveracr@purdue.edu (C.M.R.-C.); 2 The Interdisciplinary Biomedical Sciences Program—The Weldon School of Biomedical Engineering, Purdue University, West Lafayette, IN 47907, USA; 3 Purdue Center for Cancer Research and Purdue Institute for Drug Discovery, Purdue University, West Lafayette, IN 47907, USA

**Keywords:** Interleukin-27, targeted Nanoluc-IL27, Nluc-27, prostate-cancer cells, bone cells, cytokine

## Abstract

An emerging approach in treating skeletal malignancies utilizes osteoimmunology to investigate new multifunctional immune-stimulatory agents that can simultaneously combat tumor growth and promote bone repair. We have hypothesized that cytokine Interleukin-27 (IL-27) is an excellent candidate biologic to help rebalance the prostate tumor cells and bone cell environment. In this work, we examined the proof of principle for a short, secreted luciferase (Nanoluc or Nluc) fusion with IL-27 to produce a novel cytokine-based biologic (Nluc-27), whereby we examined its efficacy in vitro in reducing prostate tumor growth and rebalancing bone cell proliferation and differentiation. This work demonstrates the targeting and anti-tumor efficacy of the Nluc-27 fusion cytokine in cancer and bone cell models. The fusion cytokine is detectable in conditioned media, and bioactive in different cell systems. This novel Nluc-27 cytokine will allow flexible incorporation of other targeting domains and may serve as flexible tool to augment IL-27′s bioactivity and reengineer its efficacy against prostate tumor or bone cells, and may prove applicable to several other cell types for targeted gene therapy applications.

## 1. Introduction

Interleukin (IL)-27 can serve as a therapeutic agent for malignant tumors based on its important role in immunomodulation [1] and its relatively low toxicity [2]. IL-27 is a member of the IL-12 cytokine family and is comprised of two subunits, IL-27p28 and Epstein–Barr virus-induced gene 3 (EBI3). IL-27 signals through the IL27RA (WSX1) and IL6ST (gp130) receptor pair. IL-27 signaling induces T-box transcription factor 21 (Tbx21) and Interferon gamma (IFNγ), promoting the initiation of T-helper (Th)1 differentiation [3]. IL-27 also has direct transcriptional effects on several cell types, including tumor cells [4], and is able to induce natural-killer (NK) and cytotoxic T-lymphocyte responses, while reducing angiogenesis through CXCL9-10 upregulation [5]. We have also shown a role of IL-27 in impacting bone-cell differentiation and proliferation [6]. We previously examined a first-generation IL-27 targeted at the C-terminus with a short ‘peptide L’ (pepL, LSLITRL), which binds the interleukin 6 receptor α (IL-6Rα) that is upregulated in tumor cells [7] in order to reduce prostate tumor growth (IL-27pepL or 27pepL) [4,8,9].

In this report, we examined an enhanced version of the IL-27 molecule containing a flexible linker at the C-term, thereby enabling better accessibility of the peptide ligand for IL6Rα inhibition. Overall, we have found that the linker alteration may enable us to flexibly incorporate this approach as a novel tool to augment a cytokine’s bioactivity and efficacy against prostate tumor-cell growth.

An emerging approach in treating skeletal malignancies utilizes osteoimmunology to investigate new multifunctional immune-stimulatory agents that can simultaneously combat tumor growth and promote bone repair. IL-27 is the only member on the IL-6 family to signal via a STAT1-dominant signaling pattern [10]. Consequently, the IL-27 signaling pattern is a natural antagonist of IL-6 oncogenic signaling, leading to the activation of proinflammatory genes and the mediation of tumor-suppressive signals [11,12]. IL-27 can also serve as an effective bone-normalization agent due to its impact on pro-osteogenic-gene changes in both osteoblasts and osteoclasts [6]. Additionally, IL-27 exhibits immunomodulatory activity capable of promoting the accumulation of tumor-clearing effector cells at the site of prostate-cancer bone metastases [4,9].

Given the above-mentioned desirable properties in one multifunctional therapeutic agent, we selected IL-27 as our cytokine therapeutic to treat PCa bone metastases, and further enhanced its therapeutic efficacy through the addition of a tumor-targeting and IL-6Rα-antagonistic motif “pepL” (LSLITRL) [7]. In this work, IL-27 was fused to a secreted Nanoluciferase (Nanoluc or Nluc) that incorporates a strong secretion motif adapted from the IL-6 signal peptide, producing the ‘second-generation’ IL-27. The Nanoluc is a variant of the secreted Gaussia luciferase enzyme [13]. This fusion of a tumor-targeted cytokine with a luciferase reporter is novel because prior cytokine imaging (in vitro or in vivo) has only been achieved through radiolabeling [14,15]. Our targeted IL-27 cytokine deploys a bispecific-anchored design to effectively target PCa tumor cells with the pepL module while inducing simultaneous anti-tumorigenic and pro-osteogenic responses through the pepL and IL-27p28 modules, respectively.

## 2. Materials and Methods

### 2.1. Vectors

PCR cloning was utilized to clone the mouse IL-27elasti fragment from pUNO1-mIL-27(ebi3p28) (InvivoGen. San Diego, CA, USA; puno1-mil27) with a 3′ insertion of a sequence-encoding peptide linker (GGGGS) followed by the sequence for the targeting peptide (pepL: LSLITRL) [7], scrambled control (Scr-1: IRSLTLL), or irrelevant-peptide control (Irr-1: SIFSSHM) [16]. pNLF1-secNluc (Promega; Madison, WI, USA, N1371) was used as the vector backbone and was linearized using EcoRI. NEBuilder HiFi DNA-assembly cloning kit (New England Biolabs, Ipwich, MA, USA; E5520S) was used to generate plasmids with the secreted fusions of Nanoluc (Nluc) and targeted IL-27 as expression vectors. The Nlucs generated were Nluc-27.scr1, Nluc-27.irrel1, and Nluc-27.pepL. All vectors were prepared using GeneJET plasmid midiprep kits (ThermoFisher; Waltham, MA, USA, K0482).

### 2.2. Targeted IL-27-Conditioned Media

Targeted IL-27 was expressed in mouse myoblast C2C12 cells to model cytokine secretion from skeletal muscle following gene-therapy delivery. A modified transfection protocol utilized C2C12 cells suspended in DMEM media and transfected with IL-27 plasmids using lipofectamine 2000 (L2000) (ThermoFisher; #11668019) following manufacturer’s recommendations. Briefly, 2 µL of L2000 per 1 µg plasmid DNA were used to deliver targeted IL-27 plasmids to C2C12 cells. pIL-27/L2000 complexes were added to a 6-well plate then suspended C2C12 cells (1 × 10^6^ per well) were gently mixed with the complex and allowed to adhere and transfect for 24 h (37 °C, 5% CO_2_). L2000-containing media was then aspirated and replaced with complete DMEM media (10% FBS, 1% antibiotic and antimycotic (anti/anti, Gibco)) to allow cells to recover overnight. Following recovery, DMEM media was aspirated and replaced with OptiMEM medium (Corning, Corning, NY, USA,) to collect secreted IL-27 over 24 h. Following 24 h incubation with IL-27-transfected C2C12 cells, this OptiMEM was referred to as IL-27-conditioned media (CM). A mouse IL-27p28 Quantikine ELISA kit (R&D systems, Minneapolis; MN, USA, M2728) was used to quantify secreted IL-27 in CM. Additionally, luminescence of IL-27 CM was measured using the Renilla luciferase-assay system, which has the same substrate as Nluc (coelenterazine) (Promega; Madison, WI, USA, E2810) 

### 2.3. Characterization of Target-Cell Receptors

FACS analysis was used to characterize the target-receptor densities of cultured cells. Cells at 80–90% confluency were collected using 2 mM EDTA, washed with 1 × PBS, then resuspended in cell-staining buffer (BioLegend, San Diego, CA, USA; 420201). All samples were prepared with ~1 × 10^6^ total cells in a 100 µL final volume. For the detection of IL-6Rα, 5 µL of PE-conjugated human IL-6Rα antibody (BioLegend, San Diego, CA, USA; 352803) were added to each test sample and incubated at 4 °C for 30 min to 1.5 h. To measure IL-27Rα cell-surface density, we used 1 µg/mL of primary unconjugated human IL-27Rα (WSX1) antibody (R&D systems, Minneapolis, MN, USA; MAB1479-SP) followed by 0.25 µg of APC-conjugated anti-mouse IgG2b. An Attune NxT flow cytometer (ThermoFisher) with software version 4.2 was used to evaluate target-receptor densities. All FACS data analyses were carried out using FlowJo 10.7.1 software (Becton Dickinson, Frank Lakes, NJ, USA).

### 2.4. IL-27 Ligand-Binding Assay

HEK293 cells were cultured in complete DMEM media (10% FBS, 1% anti/anti) at 37 °C and 5% CO_2_ and split at 80–90% confluency. THP-1 cells were cultured in complete RPMI-1640 media (10% FBS, 1% anti/anti) supplemented with β-mercaptoethanol to a final concentration of 50 µM at 37 °C and 5% CO_2_ and split at 80–90% confluency. HEK293 cells were seeded at 1.25 × 10^5^ per well and THP-1 cells were seeded at 5 × 10^5^ per well in poly-L-lysine coated 24-well plates (Corning, Corning, NY, USA; 3337), then incubated at 37 °C and 5% CO_2_ overnight. Complete media was aspirated and replaced with targeted IL-27 CM, then incubated at 37 °C and 5% CO_2_ for 16 h. Following incubation with targeted IL-27 CM, cells were collected and centrifuged at 1500 rpm for 5 min to form pellets. Cells were lysed with 40 µL 1× Renilla lysis buffer, lysates were kept on ice, then 50 µL Renilla substrate were added and luminescence was measured using GloMax plate reader (Promega) with 10 sec integration time. All results are reported relative to total protein measured using a Pierce^TM^ BCA protein-assay kit (ThermoFisher; #23225) following the manufacturer’s protocols.

### 2.5. STAT-1 and STAT-3 Reporter Assays

In a 24-well plate, 2 × 10^5^ PC3 and C4-2B cancer cells were seeded into each well and allowed to adhere overnight. Lipofectamine 2000 (ThermoFisher; 11668019) was used to deliver 1 µg of plasmids responsive to active (phosphorylated) STAT-1 (pGAS/ISRE Luc) (Signosis; Santa Clara, CA, USA, LR-2016) or STAT-3 (pSTAT-3-Luc) (Signosis; LR-2004) following the manufacturer’s protocols. Transfection media was aspirated, cells were washed with 1 × DPBS, then cells were allowed to recover overnight in complete RPMI media (10% FBS, 1% anti/anti) 37 °C and 5% CO_2_. Transfected PC3 cells were serum starved for 5–6 h by replacing complete RPMI media with OptiMEM media (Corning Life Sciences; Corning, NY, USA). Following serum starvation, PC3 cells were treated with targeted Nluc-27.pepL CM for 16 h, while C4-2B were treated for 35 h. Cells were trypsinized then centrifuged at 1500 rpm for 5 min to form pellets. Cells were then lysed with 40 µL 1× passive-lysis buffer, lysates were kept on ice, then 50 µL luciferin substrate were added, and luminescence was measured using a GloMax plate reader (Promega) with 10 s integration time.

### 2.6. Prostate Cancer-Cell Proliferation

C2C12 cells were transfected with Nluc.IL-27 plasmids or an empty vector (pORF9-0) control following the procedure described above. PC3 or C4-2B cells were seeded atop a 0.4 µm PET membrane transwell (Corning Life Sciences; 353095) and allowed to adhere overnight concurrently with the post-transfection overnight recovery step of the C2C12 cells. Media was aspirated from transfected C2C12 and transwell PC3 or C4-2B cells, washed with 1 × PBS, then replaced with fresh medium. PC3 or C4-2B transwells were inserted atop C2C12 cells and incubated at 37 °C and 5% CO_2_ for 1, 2, and 3 days. For each day, cells were fixed with 500 µL 10% buffered Formalin for 15 min, then stained with 0.05% crystal violet/water solution for 15 min at room temperature, then washed and allowed to dry inverted. Next, 250 mL of 33% glacial acetic acid were used to extract the dye from the cells, then 5 µL from each well were added to 150 µL of water in a 96-well-plate format. The absorption was measured at 600 nm using a GloMax plate reader (Promega). Results are reported relative to pORF9-0 control.

### 2.7. Bone-Cell Differentiation

Osteoclast differentiation. To test cell fusion and differentiation into mature multinucleated cells (MNCs) or osteoclasts in response to treatments, 1–1.5 × 10^5^ RAW 264.7 cells (ATCC, Manassas, VA, USA) were seeded in a 12-well plate. Cultured cells were treated with RANKL (35 ng/mL) alone, RANKL + Nluc-27 (scr1, irrel1, or pepL), or no treatment control for 6 days. Cells were stained for detection of the marker protein tartrate-resistant acid phosphatase (TRAP) (Cosmo Bio USA; Carlsbad, CA, USA) following manufacturer’s protocols. The area of MNCs relative to total area was measured in 5–10 independent high-power microscopic fields and reported as an average of area covered by MNCs relative to total area.

### 2.8. Osteoblast Differentiation

Pre-osteoblast MC3T3E1(clone 14) or MC3T3E1(14) cells (ATCC) were used to assess targeted Nluc-27.pepL’s ability to promote osteoblast differentiation relative to Nluc-27 controls (scr1 or irrel1), with each being secreted into the conditioned media. MC3T3E1(14) cells were cultured in 10% heat-inactivated FBS (ATCC)/α-MEM media (ThermoFisher) with 1% anti-anti. Next, 1–1.2 × 10^5^ MC3T3E1(14) cells per well were seeded into a 48-well flat-bottom plate and cultured with differentiation supplements (Millipore; ECM810, ascorbic acid and 2-glycerol phosphate) or without differentiation supplements. FBS was heat inactivated by incubation at 55 °C for 30 min, then kept at 4 °C prior to use. Both cell types were treated with either targeted mIL-27pepL CM or controls. MC3T3E1(14) cell differentiation was determined by RT-qPCR for early osteoblast-differentiation genes (osteocalcin (OCN); bone sialoprotein (BSP); and collagen I A1) at day 7. Two additional controls were used in this assay: No treatment (NT), in which osteoblasts did not receive any treatment or conditioned media, and media+ in which osteoblasts received differentiation induction treatments and were not supplemented with conditioned media.

### 2.9. Statistical Analysis

Assays were performed in triplicate and values are provided as mean ± SEM. Comparisons were performed using unpaired *t*-tests or one-way analysis-of-variance analyses (one-way ANOVA). A *p*-value < 0.05 indicates a significant difference (*). 

## 3. Results

### 3.1. In Silico Predictions and Structural Alignment of Targeted IL-27

**Figure 1:**
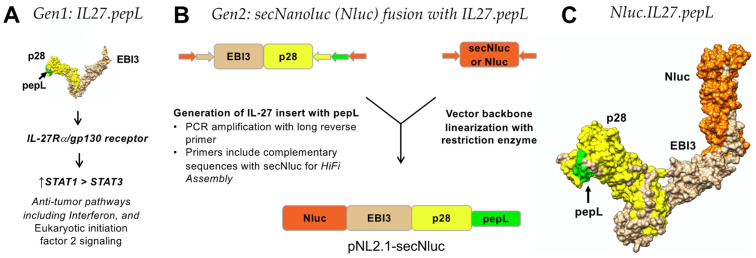
Development of IL-27 chimeric cytokines. (**A**) Generation 1 (Gen1) of IL-27 (IL27p28 and EBI3 subunits) with pepL at the C-terminus. Antitumor pathways listed briefly summarize the signaling effects reported in [8]. (**B**) Generation 2 (Gen2) of the IL27.pepL consists of its fusion at the N-terminus to a secreted Nanoluc (Nluc). Shown is the vector evolution of targeted Nanoluc-27 (Nluc-27.pepL). Mouse IL-27elasti fragment from pUNO1-mIL-27(ebi3p28) (InvivoGen) with a 3′ insertion of a sequence-encoding peptide linker (GGGGS) (not shown) followed by the sequence for the targeting peptide (pepL: LSLITRL), lime green; scrambled control (scr1: IRSLTLL), not shown; or irrelevant-peptide control (irrel1: SIFSSHM), not shown. pNLF1-secNluc (Promega) was used as the vector backbone and was linearized using EcoRI. NEBuilders HiFi DNA-assembly cloning kit (#E5520S) was used to generate plasmids with the fusions of Nluc and targeted IL-27-expression vectors. (**C**) In silico modeling of the final construct of Nluc-27 (I-TASSER) showing the targeting module (pepL) is solvent exposed (lime green).

Prior work from our group has indicated that a first-generation IL-27 (Gen 1; Figure 1A) could be modified at the C-terminus with a short peptide that enhanced its therapeutic efficacy. This peptide consisted of a tumor-targeting and IL-6Rα-antagonistic motif, “pepL” (LSLITRL) [7]. In this work, we sought to further enhance IL-27 by fusing it to a secreted Nanoluciferase (Nanoluc) that incorporates a strong secretion motif adapted from the IL-6 signal peptide, producing the second-generation IL-27 (Gen 2; Figure 1B). This Gen 2 targeted IL-27 cytokine deploys a bispecific-anchored design to effectively target PCa tumor cells with the pepL module while inducing simultaneous anti-tumorigenic and pro-osteogenic responses through the pepL and IL-27p28 modules, respectively (Figure 1C).

**Figure 2:**
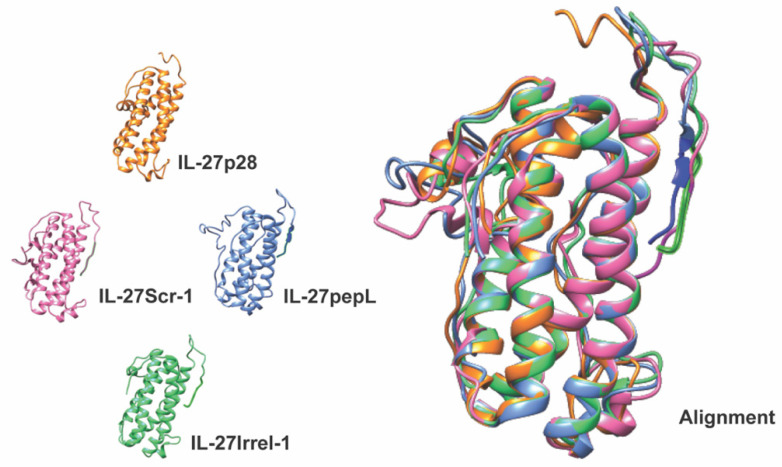
*In silico* modeling and alignment shown for the most-distal C-terminal portion of Nluc-27, consisting of the native IL27p28 subunit, a short linker (GGGGS), and each of the control (scr1 or irrel1) or targeted (pepL) peptides. All C-terminal peptide sequences used were predicted to be solvent exposed.

To examine whether modification of the IL-27p28 subunit with the targeting peptide (pepL) or control sequence peptides (scr1, scrambled-1, or irrel1, irrelevant-1) might impact the native secondary structure of IL-27p28, we utilized I-TASSER modeling focusing on the most distal N-terminal portion of the fusion protein (Figure 2). This approach enabled us to predict and align the secondary structures to a native IL-27 model. Most importantly, structure prediction guided our selection of control peptides. All peptides were solvent accessible, thus any results reported are due to sequence and therapeutic-efficacy differences of IL-27 modified with peptides at the C-terminus. 

### 3.2. Secreted Nluc-27 Fusion Proteins Retain Functional Activity in Conditioned Media

**Figure 3:**
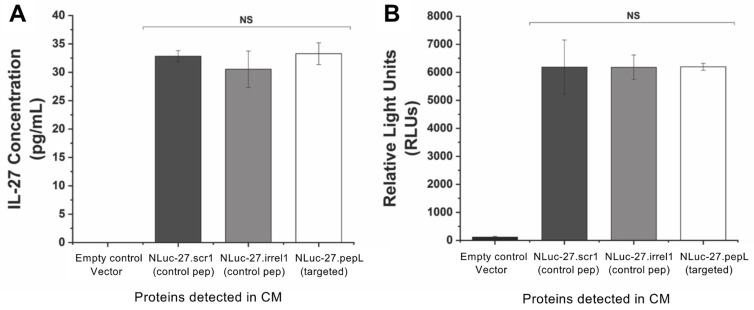
Expression-level analysis of secreted Nluc-27 molecules in conditioned media with (**A**) quantitative ELISA of IL-27p28 in pg/mL, and (**B**) luciferase activity reported as relative light units (RLUs/s) of NLuc fusion IL-27 using the Renilla luciferase assay (Promega). Both assays indicate the final fusion products are active with equivalent expression levels. The correlation coefficient was calculated to be 0.995. Data are presented as mean ± SEM. Comparisons were performed using one-way ANOVA. A *p*-value ≥ 0.05 indicates a non-significant difference (NS).

We also measured the expression/secretion of our targeted or control Nluc-27 fusion proteins in conditioned media collected from transfected C2C12 cells by using quantitative ELISA (Figure 3A) and relative luminescence (Figure 3B). Both measurements detected that the expression levels of the targeted or control Nluc-27 fusion proteins were expressed at equivalent levels, indicating that the treatment effects observed in this study were not due to variations in cytokine CM concentrations.

### 3.3. Variable Receptor Densities on Surface of Model Cell Lines

**Figure 4:**
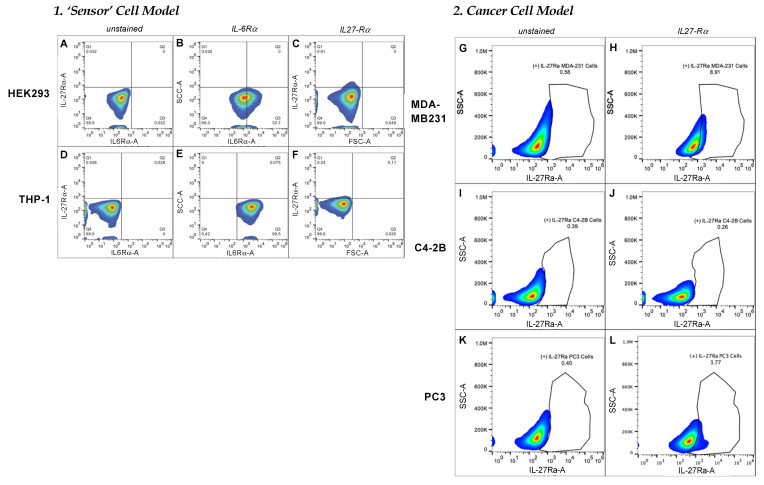
IL-6Rα- and IL-27Rα-expression profile on surfaces of cells utilized as reporter or ‘sensor’ cells to validate 2nd-generation Nanoluc-27 targeting. Both (**A**,**D**) are unstained controls of sensor cells. Around 30% of HEK293 cells displayed IL-6Rα, whereas ~90% of THP-1 cells displayed the receptor on their cell surface in measurable densities (**B**,**E**). Both HEK293 and THP-1 displayed negligible densities of IL-27Rα (**C**,**F**). 2. IL-27Rα-expression profile on surface of cancer-cell models. (**G**,**I**,**K**) are unstained controls of model cells. IL-27Rα-expression levels in MDA-MB231 (positive control) at 8.91% (**H**), C4-2B cells at 0.26% (**J**), and PC3 cells at 3.77% (**L**).

To characterize the IL-6-receptor-alpha (IL-6Rα)- and the IL-27-receptor-alpha (IL-27Rα)-subunit densities on several types of potential target cells, we utilized fluorescence-activated cell sorting (FACS) (ThermoFisher Attune NxT) with two different cell models (Figure 4). One was a ‘sensor-cell’ model, where non-cancerous cell lines were selected that have been reported to express either low or high levels of IL6-Rα, thus are able to sense signaling changes downstream of that receptor. Another was a ‘cancer-cell’ model, where tumor cell lines were selected that expressed variable levels of IL-27Rα. 

For the first model examined, HEK293 exhibited moderate IL-6Rα expression in ~30% of the cells (Figure 4B) relative to the control (Figure 4A), which was consistent with the Human Protein Atlas (HPA) [17]. Conversely, 100% of THP-1 cells expressed IL-6Rα (Figure 4E), which was also supported by the HPA. Surprisingly, the expression levels of IL-27α on HEK293 or THP-1 cell surfaces were negligible. 

For the second model examined, when we assessed the expression of IL-27α on the surface of prostate-cancer cells (PC3 and C4-2B) only negligible levels were detected, with 3.77% of PC3 and 0.26% of C4-2B cells displaying the receptor relative to their unstained controls (Figure 4I–L). We utilized MDA-MB231 as a positive control since it is a breast-cancer cell line that is reported to overexpress IL-27Rα on the cell surface. However, our results showed that only 8.91% of these cells displayed measurable densities of IL-27Rα relative to its unstained control (Figure 4G,H).

**Figure 5:**
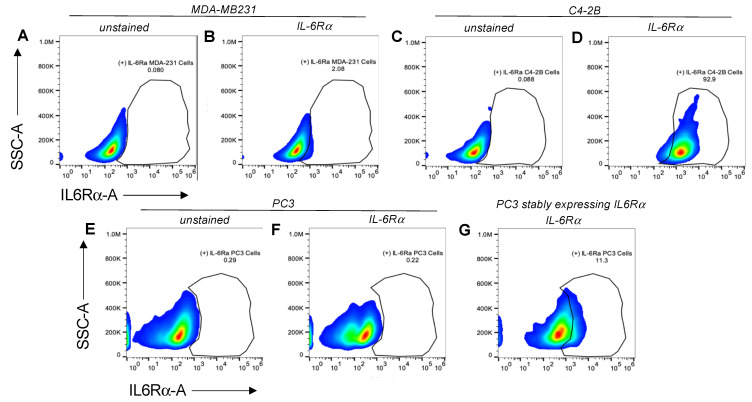
IL-6Rα-expression profile on the surface of cells utilized in STAT-1/-3-activation assays. MDA-231 cells (**B**) serve as a model of cell lines with ‘lower levels’ of the receptor. (**A**,**C**,**E**) are unstained controls of tested cell lines. More than 90% of C4-2B cells (**D**) expressed measurable levels of IL6-Rα. PC3 cells expressed negligible levels of IL-6Rα (**F**), while ~11% of PC3 cells stably expressing an IL6Rα construct (plasmid transfection and antibiotic selection) showed detectable levels of the receptor (**G**).

We next assessed the expression of IL-6Rα on the surface of the cells that we planned to utilize in STAT1/3-activation assays, as reporters or ‘sensors’ of Nanoluc-27 signaling. IL-6Rα was detected at low levels in MDA-MB231 and PC3 cells (<2%; Figure 5B,F) relative to the unstained controls (Figure 5A,E), whereas its expression in C4-2B prostate-cancer cells reached ~93% (Figure 5D) relative to its unstained control (Figure 5C). PC3 cells stably expressing a plasmid expressing IL-6Rα reached ~11% of IL-6Rα positive cells (Figure 5G) relative to the unstained control (Figure 5E). Together, these cells represent potential models with low and high levels of the IL-6Rα receptor for examining signaling downstream of Nanoluc-27 in vitro.

### 3.4. Targeted Nluc-27 Is Able to Activate the STAT-1 Signaling Pathway in Cells Expressing IL-6Rα

**Figure 6:**
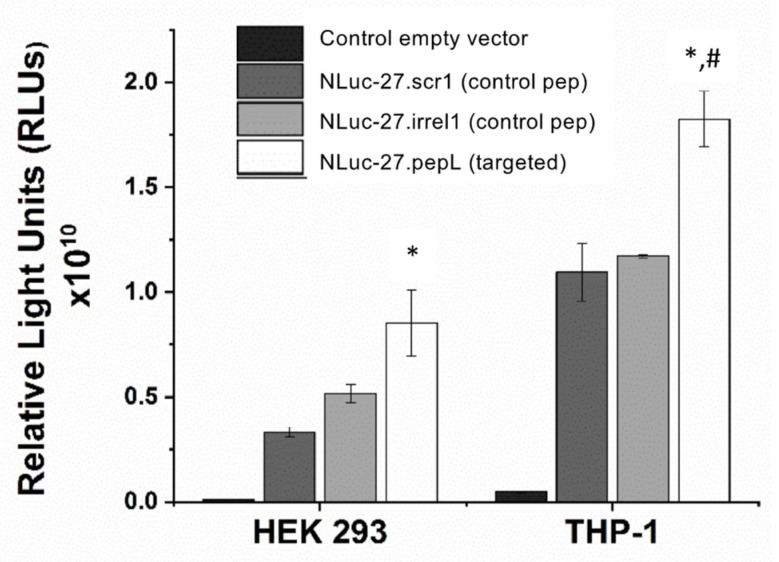
Validation of ligand-targeting efficacy in ‘sensor cells’ displaying differential expression of the target cytokine receptor, IL-6Rα. Targeted Nluc-27pepL displayed a signal significantly higher than controls when incubated with HEK293. This ligand binding (homing) was enhanced in THP-1 cells which presented significantly more IL-6Rα on the cell surface. Data are presented as mean ± SEM. Comparisons within cell types were performed using one-way ANOVA. A *p*-value < 0.05 indicates a significant difference (*). Comparisons between cell types were performed using unpaired *t*-test. A *p*-value < 0.05 indicates a significant difference (#).

To assess binding of Nluc-27 to the proposed targeted receptor (IL-6Rα) (Figure 6), we used HEK293 and THP-1 cells, which we have characterized as displaying differential expression of this cell-surface receptor (Figure 4). All peptide-modified Nluc-27 species exhibited binding to both HEK293 and THP-1 to some extent. However, modification with pepL enhanced the Nluc-27 signal accumulation in both HEK293 and THP-1, with significantly greater binding of Nluc-27pepL to THP-1, which is a cell line with higher receptor density.

**Figure 7:**
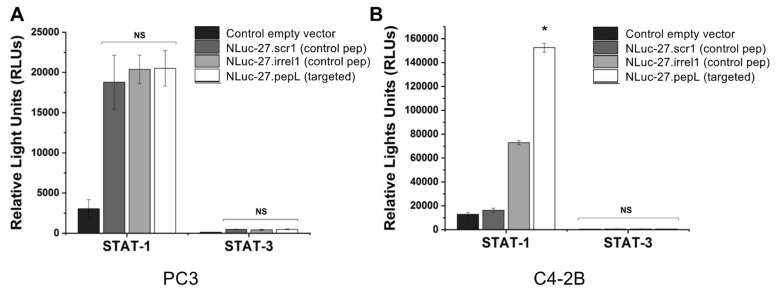
STAT-1 and STAT-3 activation in PC3 and C4-2B prostate ‘cancer-cell’ models following treatment with Nluc-27-conditioned media. (**A**) Treatment with targeted Nluc-27pepL in PC3 cells induced STAT-1 activation in response to targeted (pepL) and non-targeted Nluc-27 (Scr-1, Irrel-1), with minimal activation of STAT-3 (non-significant, ns). (**B**) Treatment with targeted Nluc-27pepL in C4-2B cells induced a 12-fold in STAT-1 activation relative to control vector, with no significant changes (ns) in STAT-3 expression in response to treatment with Nanoluc-27 (targeted or non-targeted). Data are presented as mean ± SEM. Comparisons within cell types were performed using one-way ANOVA. A *p*-value < 0.05 indicates a significant difference (*).

We also conducted functional assays to evaluate the anti-tumorigenic and pro-apoptotic potential of our therapeutic (targeted Nluc-27pepL) (Figure 7). Treatment with all Nluc-27 fusion proteins (pepL or peptide controls: scr-1 and irrel-1) significantly upregulated STAT-1 expression in PC3 cells transfected with a STAT-1 luciferase reporter (pGAS/ISRE-Luc), indicating that all Nluc-27s retained the full functionality of the IL-27 portion of the fusion proteins. STAT-3 activity was slightly increased following treatment with the various Nluc-27 species, but this difference was not significant. This is expected due to overlapping signaling factors, and homo- or hetero-dimerization of the signal transduction subunit with IL-6 family receptors. However, the targeting moiety (pepL) and Nluc-27 appear to primarily promote a pattern of STAT-1 activation.

### 3.5. Targeted Nluc-27 Reduces Cancer-Cell Proliferation

**Figure 8:**
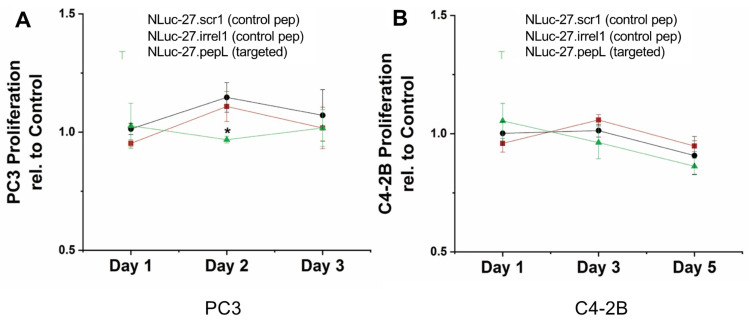
Effect of targeted Nluc-27.pepL-conditioned media on cancer-cell proliferation. (**A**) Cells that displayed moder-ate levels of IL-6Rα, PC3 cells, and (**B**) cells that overexpressed IL-6Rα, C4-2B cells. Only the cells treated with targeted Nluc-27.pepL exhibited a trend in the reduction in cell proliferation relative to the empty-vector control group. Data are presented as mean ± SEM. Comparisons within cell types were performed using one-way ANOVA. A p-value < 0.05 indi-cates a significant difference (*). Red squares, Nluc-27.scr1 (control peptide), black circles, Nluc-27.irrel1 (control peptide), and green triangles, Nluc-27.pepL (targeted).

To evaluate the therapeutic potential of targeted Nluc-27.pepL, we assessed changes in prostate-cancer-cell numbers over time in vitro as an estimate of prostate-cancer-cell proliferation. This assay was performed in transwell cultures with C2C12 mouse myoblast cells transfected with different plasmids to express either Nluc-27 with C-term control peptides (scr1, irrel1) or Nluc-27.pepL (targeted) fusion proteins. PC3 cells cultured in Nluc-27.pepL-conditioned media (CM) exhibited a reduction in cell proliferation compared to the controls (Figure 8). Similarly, C4-2B cultured in Nluc-27.pepL CM showed reduced cell proliferation compared to the controls. The reduction in cell proliferation was likely more prominent in C4-2B due to their higher density of the target receptor (IL-6Rα). Although a pattern of reduced cell proliferation was only significant at day 2 of the PC3 assays (Figure 8A), treatment with targeted Nluc-27.pepL promoted a more sustained trend of reduced C4-2B proliferation throughout the experiment (Figure 8B), although this difference did not reach significance.

### 3.6. Targeted Nanoluc-27pepL Promotes Bone Cell Differentiation

**Figure 9:**
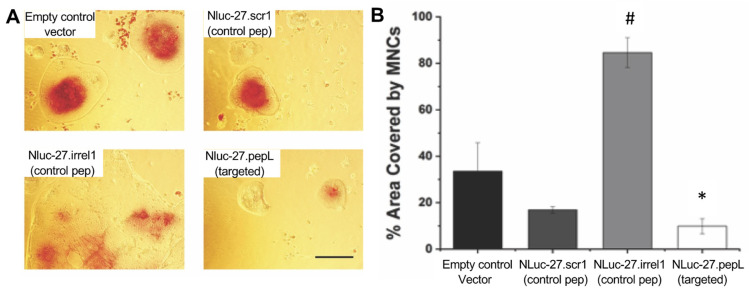
Targeted Nluc-27.pepL inhibits RANKL-mediated osteoclast formation in pre-osteoclastic cell line RAW 264.7. (**A**) NLuc-27.pepL significantly reduced the size of osteoclasts formed compared to the control empty vector or Nluc-27.scr1 (*, *p* < 0.05). (**B**) The Nluc-27.irrel1 control induced significantly larger multinucleated cells (MNCs) compared to the other three groups (#, *p* < 0.05). Comparisons were performed using unpaired *t*-test. Scale bar 100 μm.

To evaluate the therapeutic potential of targeted Nluc-27.pepL in rebalancing bone remodeling, we examined bone-cell differentiation in response to the different Nluc27 treatments. Pre-osteoclastic RAW 264.7 cells (Figure 9) were treated with CM containing control Nluc-27 (scr1 or irrel1) or targeting (pepL) peptides. Compared to the empty-plasmid control (pORF9-0), Nluc-27.scr1 and Nluc-27.pepL both significantly reduced the area covered by osteoclast-like multinucleated cells (MNCs) that formed in the presence of RANKL. The targeted NLuc-27.pepL further reduced the area covered by MNCs. On the other hand, CM containing NLuc-27.irrel1 induced MNCs that were two-fold the size of the control osteoclasts that were treated with the empty-plasmid control (pORF9-0), which was an unexpected finding (Figure 9).

**Figure 10:**
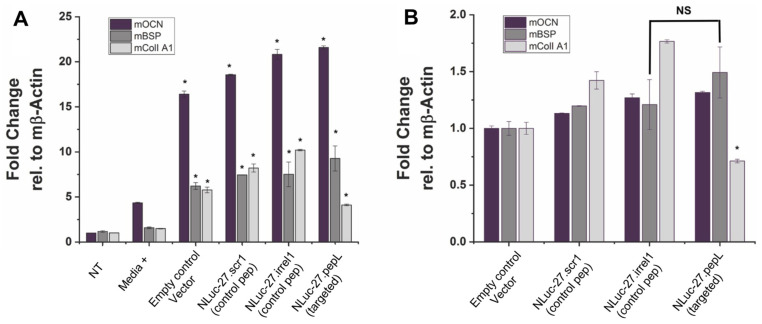
Targeted Nluc-27 upregulates genes associated with osteoblast differentiation in the mouse MC3T3-E1 (Subclone 14) pre-osteoblastic cell line. (**A**) Relative to no treatment (set at 1.0) and positive control (media+; containing differentiation supplements), all CM induced higher levels of the three osteoblast-differentiation genes assayed (*, *p* < 0.05). Shown are fold changes in gene expression by qPCR relative to mouse β-actin. (**B**) Relative to control empty vector *CM* (set at 1.0), moderate upregulation of osteoblastic-differentiation genes was detected in response to NLuc-27.scr1 or irrel1, but this difference was not significant (ns). Shown are fold changes in gene expression by qPCR relative to mouse β-actin. Changes in mBSP trended higher for Nluc-27.pepL relative to control and Nluc-27.irrel1, but the only significant change for Nluc-27.pepL was a reduction in the levels of Col IA1 relative to the control Nluc-27s (scr1, and irrel1)(*, *p* < 0.05). All gene-expression analyses were from MC3T3-E1-14 cells isolated at day 7 of treatment. Data are presented as mean ± SEM. Comparisons were performed using one-way ANOVA.

We also evaluated the effects of targeted NLuc-27.pepL on genes associated with osteoblast differentiation (mouse osteocalcin, mOCN; bone sialoprotein, mBSP; and collagen IA1, mColI A1). The negative control (NT) and positive control (media+, containing differentiation supplements) exhibited the expected profile of gene expression in differentiating-osteoblasts gene expression (Figure 10A). All groups treated with conditioned media (CM), including the empty-plasmid control (pORF9-0), exhibited a significant increase in the expression of all three genes related to osteoblast differentiation compared to the positive control (media+). Treatment with NLuc-27s CM resulted in a moderate yet statistically significant increase in osteoblast-differentiation gene expression, with targeted NLuc-27.pepL inducing a trend of higher expression of OCN and BSP relative to the empty-plasmid control (Figure 10A,B). ColIA1 expression in cells treated with Nluc-27.pepL was significantly lower than in cells treated with CM from the controls (control pORF9-0 or empty vector control, NLuc-27.scr1 or irrel1), yet significantly higher than ColIA1 expression in cells treated with media+.

## 4. Discussion

In the present work, we validated the targeting and bioactivity of our second-generation IL-27 therapeutic, which is a fusion of a secreted Nanoluc (Nluc) with IL-27 that was modified at the C-terminus with targeting peptide pepL (Nluc-27.pepL). Earlier work in our lab explored the anti-tumorigenic and pro-osteogenic profiles induced by IL-27 and discovered that IL-27pepL stimulated the expression of luc reporters of STAT-1 and IFNγ in vivo [4]. The second-generation targeted IL-27 described in this manuscript is novel since it preserves the bioactivity of the mouse IL-27 while enabling the cytokine to be detected via fusion with the small reporter molecule, Nluc. This new feature produced a secreted Nluc-IL27 or Nluc-27, which allows us to assess the activity of IL-27 in cells in real time. To our knowledge, this approach is novel in cytokine research and has not been previously reported. We conducted a series of secondary-structure predictions to ensure that the proposed targeted Nluc-27 constructs could be targeted via the solvent-exposed C-terminus peptides, yet without destabilizing the structure of the IL-27p28 subunit, thus avoiding detrimental impacts on its cytokine activity. We utilized ELISA analysis to quantify secreted Nluc-27 levels for both the targeted and control forms. We also tested the Nanoluciferase activity of our Nluc-27 fusions to ensure that the addition of IL-27 did not change its activity as a reporter protein. Both approaches informed us that all forms of secreted Nluc-27 retained their biological activity. 

To examine the targeting ability of the Nluc-27.pepL, we utilized two cell lines (HEK293 and THP-1) that have been identified as presenting different levels of the target receptor (IL-6Rα) as reporter or ‘sensor’ cells. Following treatment, the data indicated that targeted Nluc-27.pepL bound to HEK293 cells at significantly higher levels than the non-targeted fusion-protein forms. The homing levels of targeted Nluc-27.pepL were further enhanced in THP-1 cells, suggesting that this protein might be able to accumulate at prostate tumors expressing the targeting receptor IL-6Rα. Next, we investigated the ability of the targeted Nluc-27.pepL to activate STAT-1/-3 pathways in cancer cell lines. At first, we tested the ability of mouse IL-27 fusion to activate STAT-1 and inhibit STAT-3 in PC3 cells. We found that all ‘mouse’ IL-27 forms could activate STAT-1 in PC3 cells without significantly activating STAT-3. Here, however, we did not observe any enhanced effect in STAT-1 activation as a function of the targeting modality. We concluded that the cross-species activation of the STAT-1 pathway is possible for human IL-6Rα and IL-27Rα with mouse isoforms; however, the full desired benefit of enhanced activation to achieve therapeutic levels is only possible with species matching. Many of the cell models used in research are underdeveloped or not suited for varying approaches in therapeutics development. For example, the PC3 cell line is widely used for research in bone metastases of prostate-cancer research. This cell line is representative of the aggressiveness of metastases but does not appropriately model the effect of metastases on bone (osteoclastic vs. osteoblastic lesions). Furthermore, available STAT-1/-3 reporters are commonly designed for human cells, with a severe lack of comprehensive mouse-cell models/reporters. We thus adapted the assays and vectors to also be able to examine the impact of Nluc-27 on signaling. By treating prostate-cancer cells (C4-2B) transfected with STAT-1/-3 reporters (pGAS/ISRE and pSTAT-3Luc, respectively) with the Nluc-27 fusions, we achieved significant STAT-1 activation in cells, particularly in cells treated with Nluc-27.pepL Both Nluc-27.scr1 and irrel1 untargeted forms also induced STAT-1 activation due to the native activity of IL-27. However, the targeting modality (pepL), which also serves as an IL-6Rα antagonist, amplified STAT-1 activation, thus potentially augmenting the therapeutic efficacy of current and future Nluc-27-based gene therapies.

Another key target in the development of therapeutics for treating skeletal malignancies is to aim for the restoration of bone-remodeling balance. Two cell lines were selected for assays examining osteoclast and osteoblast differentiation: mouse pre-osteoclastic RAW 264.7 and pre-osteoblastic MC3T3-E1-14 lines. In RAW 264.7, the targeted Nluc-27.pepL significantly reduced RANKL-mediated osteoclast formation. The results were reported relative to the total field of view due to the large size of osteoclasts formed in this assay and were compared to controls (empty plasmid and Nluc-27.scr1). Interestingly, treatment with Nluc-27.irrel1 induced significantly larger multinucleated cells (MNCs) compared to the controls and the targeted Nluc-27.pepL. The BLAST analysis of the irrel1 peptide (SIFSSHM) indicated a potential alignment with the LIM domain-binding protein 3. LIM proteins contain zinc finger domains composed mostly of cysteine and histidine residues. The relationship between LIM 3 and RANKL-mediated osteoclast formation has not been reported. However, a recent study on LIM 1 and osteoclastogenesis reported that LIM 1 was upregulated during RANKL-mediated osteoclast formation [18]. Additionally, knockdown of LIM 1 in a RAW-D cell line was shown to enhance the formation of MNCs and osteoclast-differentiation gene markers [18]. Therefore, it is possible that due to the structural similarities between LIM 3 and irrel1, that irrel1 (and LIM 3) might play an opposing role to that of LIM 1. This can potentially explain why, when treated with Nluc-27.irrel1, the effect of RANKL-mediated osteoclast formation was enhanced, resulting in a two-fold increase in the size of osteoclasts formed compared with the pORF9-0 control. 

In the MC3T3-E1-14 preosteoblastic cell line, the treatment with targeted Nluc-27.pepL significantly upregulated three genes associated with osteoblast differentiation (osteocalcin, Ocn or Bglap; bone sialoprotein, Bsp or Ibsp; and collagen IA1, ColI A1 or Col1a1) compared to the media+ positive control. Interestingly, all conditioned-media treatments including the treatment with the empty-plasmid control pORF9-0 promoted a significant upregulation of osteoblast-differentiation gene markers, indicating that other secreted factors from C2C12 cells also modulate osteoblast-differentiation gene markers in MC3T3-E1-14 cells. Nluc-27.pepL showed a trend of the highest upregulation of OCN and BSP, but not CollA1, and this could be due to the timing of gene expression during osteoblast differentiation. For instance, BSP is considered an early stage osteoblast-differentiation marker, while Ocn and ColIA1 are considered to be late stage osteoblast-differentiation markers [19]. We should note that this data is somewhat limited in that it utilized gene-expression assessment, and further protein analyses could be pursued in future studies to confirm these findings. Nevertheless, the literature supports the correlation between RNA and protein levels for Ocn [20], Bsp [21] and CollA1 [22] in experimental settings relating to osteoblastic differentiation. 

Relative to the pORF9-0 control, Nluc-27s (targeted and non-targeted) induced a moderate yet significant upregulation of osteoblast-differentiation gene markers, with targeted Nluc-27.pepL being the most efficient at upregulating osteoblast-differentiation gene markers. The exception here was the ColIA1 gene. ColIA1 was significantly upregulated in response to treatment with Nluc-27.pepL when compared to media+ (~four-fold), yet to a lower extent relative to the effect of Nluc-27 (scr1 and irrel1). One of the principal functions of bone tissue is to provide resistance to mechanical forces and fractures. Type I collagen is the primary organic component of the bone matrix, and mineralized collagen plays a key role in providing the bone with tensile strength. Disruptions or mutations in type I collagen in the bone matrix can lead to disorders such as increased bone calcification. With that in mind, the increase in ColIA1 gene expression in response to conditioned media (pORF9-0, Nluc-27.scr1 or irrel1) relative to media+ might not be entirely beneficial in balancing bone remodeling in interaction with tumor cells, for example. Therefore, the moderate increase in ColIA1 gene expression in response to Nluc-27.pepL could prove to be more efficacious in restoring bone homeostasis without increasing the likelihood of bone-mineralization disorders.

As a therapeutic, IL-27 is not reported in the literature to promote the direct killing of cancer cells. However, Figure 8 demonstrates some significant reduction in prostate cancer-cell proliferation in PC3 cells, and a trend of sustained decrease in proliferation of C4-2B cells, which overexpress the target receptor of Nluc-27.pepL. Although this cell-culture experiment is limited by its current design (six days), the trend of reduced proliferation with IL-27-based treatments is supported by various ongoing projects within our group. It appears likely that the full therapeutic potential of targeted Nluc-27.pepL can be best revealed in more complex assays, i.e., settings where we may be able to combine cancer and bone cells in co-culture or follow-up in vivo experiments. 

In summary, in developing the second generation of targeted IL-27 we leveraged our experience with the first generation and improved upon the original design through the fusion with Nanoluc, which is designed to enable real-time imaging of a therapeutic cytokine and its measurement in conditioned media, a novel approach that had not been previously reported. To test this design, we developed non-targeted controls to elucidate the effects of our peptide modification on the structure and activity of our cytokine therapeutic, IL-27. We also tested the targeted IL-27′s ability to home to cells expressing the target receptor and its ability to induce the desired therapeutic effects of anti-tumorigenic and pro-osteogenic pathway activation. Improvements to the delivery of these therapeutics will also continue, to be achieved by improved long-term expression vectors for gene delivery [23], by targeting the IL-27 to other conditions in other current ongoing projects, which include anti-inflammatory approaches for arthritis and for treating respiratory-distress-related conditions.

## Data Availability

Data is available upon request.
